# A Numerical Study of ITZ Percolation in Polyphase Concrete Systems Considering the Synergetic Effect of Aggregate Shape- and Size-Diversities

**DOI:** 10.3390/ma16062515

**Published:** 2023-03-22

**Authors:** Jianjun Lin, Qingxin Zhao, Huisu Chen, Mingqi Li, Lili Yuan

**Affiliations:** 1 State Key Laboratory of Metastable Materials Science and Technology, Yanshan University, Qinhuangdao 066000, China; 2 Jiangsu Key Laboratory of Construction Materials, School of Materials Science and Engineering, Southeast University, Nanjing 211189, China; 3 Hebei Province Engineering Research Center for Harmless Synergistic Treatment and Recycling of Municipal Solid Waste, Yanshan University, Qinhuangdao 066000, China; 4 School of Civil and Transportation Engineering, Hebei University of Technology, Tianjin 300401, China; 5 Shenzhen Guoyi Park Construction Co., Ltd., Research and Development Center, Shenzhen 518040, China

**Keywords:** polyphase concrete, ITZ percolation, synergetic effect, aggregate shape, numerical modeling

## Abstract

The percolation of the interfacial transition zone (ITZ) is generally regarded as an important factor that may accelerate the penetration of aggressive agents in concrete materials, and its threshold is largely determined by the features of aggregates. In most numerical studies about ITZ percolation, both fine aggregates and coarse aggregates are assumed to be the particles of uniform shape, and their size distributions are generally strung together by a single function, which is quite different from reality. To quantify the ITZ percolation associated with the polydispersity of aggregate shapes and size gradations in a more realistic way, the two-dimensional (2D) meso-scale model of concrete is generated by simplifying coarse aggregates and fine aggregates as polygons and ovals, respectively. Moreover, the size gradations of them are also represented by two separate expressions. By combining these models with percolation theory, the percolation of ITZ in the 2D case is explicitly simulated, and the influence of aggregate shape- and size-diversities on the critical threshold *ϕ*_agg,c_ is studied in detail. Based on the simulated results of *ϕ*_agg,c_, an empirically analytical expression is further proposed to fast predict the ITZ percolation, and its reliability is verified. The results show that the ITZ thickness, average aggregate fineness, coarse aggregate shape, and fine aggregate shapes are the four main contributing factors to the ITZ percolation. Compared with the existing literature, the proposed model here has a broader range of applications (e.g., mortar, concrete, and other granular systems) in the 2D case and can provide the larger predicted results, which may be closer to reality.

## 1. Introduction

The interfacial transition zone (ITZ) [1,2,3] is commonly deemed to be an important component around the aggregate particles in concrete, and its distinct characters (e.g., higher porosity, lower strength, and larger permeability) can facilitate the penetration of aggressive ions into materials and the stress concentration to some extent, which in turn leads to the degradation of material performance [4,5]. Specially, the ITZ percolation [6,7] is mainly used to reflect the global connectivity of the ITZs around the aggregates, and its critical threshold can be generally regarded as a contributing factor that determines the micro-structure and macro-properties of materials. According to the percolation theory [8,9], once the global connectivity of ITZs emerges, a dramatic and rapid change in material properties may occur. Therefore, exploring the ITZ percolation in concrete is of great significance for evaluating the effective performance of materials.

**Figure 1:**
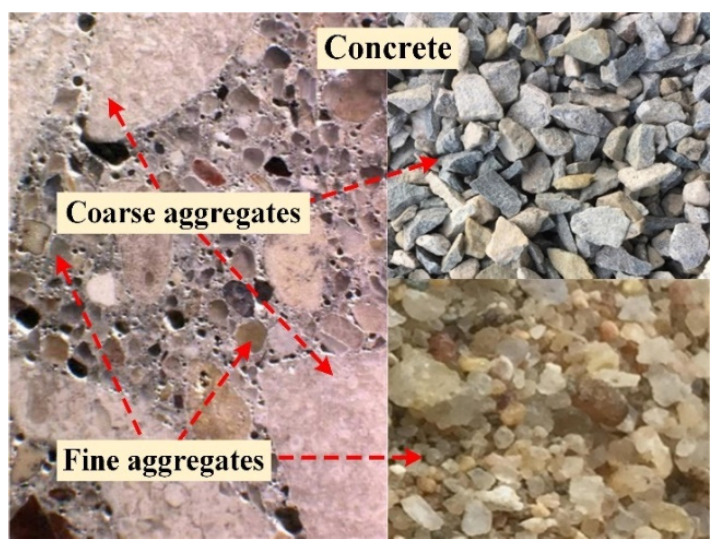
The morphologies of both coarse aggregates (i.e., gravels) and fine aggregates (i.e., sands) in actual concrete.

Due to the complexity and changefulness of the ITZ phase in concrete, it is generally difficult to experimentally identify and quantify the partial characteristics of the ITZ phase, such as ITZ thickness, relative content, percolation behavior, etc. To remedy this deficiency, computer simulation has become a powerful alternative tool in the field of percolation [10,11,12]. Up to now, a large number of researches about the ITZ percolation in concrete have been conducted by simulation, and considerable achievements have been successively obtained. For instance, by assuming the aggregate particles as regular-shaped particles (e.g., spheres [13,14,15], ellipsoids [16,17], polygons [18], polyhedrons [19], etc.) with polydisperse sizes, the sensitivity of the ITZ percolation to the aggregate shapes and sizes was studied individually, and the findings were concluded as below: (a) the ITZ thickness is the dominant factor that determines the ITZ percolation [14,15,16,17,18,19]; (b) the percolation threshold of ITZ is very sensitive to the specific surface areas (SSA) of particles and shows an obviously downward trend with the increase of SSA [17,18,19]; (c) with the increase of the aspect ratio of elongated ellipsoids [16,17] or the decrease of the number of polygon edges [18] and polyhedron faces [19], the percolation threshold of ITZ around them could be decreased significantly. All of these studies [14,15,16,17,18,19] supported the importance of aggregate shape and size in the prediction of ITZ percolation. However, there are still several problems that need to be improved because of two simplistic assumptions generally used in the previous papers. The first assumption is that both coarse aggregates and fine aggregates are commonly simplified as regular particles of uniform shape, which overlooks the geometric differences between them [15,16,18,19]. As shown in Figure 1, the aggregate particles in concrete can be roughly classified into two categories: coarse aggregates (i.e., gravels) and fine aggregates (i.e., sands). Morphologically, the geometric shapes of coarse aggregates are very similar to the regular/irregular convex polygons or polyhedrons [18,19,20,21,22,23]. By contrast, the fine aggregates look more like the non-spherical particles of smooth surfaces such as spheres [13,14,15], ellipsoids [16,17], and ovoids [7,24,25], etc. In addition, the other simplistic assumption is that both the size distributions of coarse aggregates and fine aggregates in the literature about ITZ features are generally strung together and quantitatively represented by a single function [15,16,18,19,20,21,22,23,24], which is also quite different from the reality in concrete. The oversimplification of aggregate shapes and size distributions in previous studies may result in a greater deviation between the predicted approximation and the reality.

To emphasize the effect of the shape diversity of granular components in materials, a series of complex granular systems have been applied recently in the study of ITZ percolation. For example, a combination body of ellipsoids and spheres is adopted to represent the fine aggregates and air-voids in the air-entrained mortar, and the dependence of ITZ percolation on the shape features of both aggregates and air-voids is obtained [26]. All the specific surface areas of the fine aggregate and air void, ITZ thickness, and air-void volume fraction can be regarded as crucial parameters to the ITZ percolation. In the literature [27], the fine aggregates and lightweight (LWA) aggregates are respectively assumed to be the spherical particles with the fixed thickness of ITZ and the spherical particles with no ITZ, and the sensitivity of ITZ percolation to the relative content of different types of aggregates is presented. The figures in this paper show that the volume fraction of percolated ITZ for the LWA mortar is generally lower than that for the normal mortar. In addition, by assuming the aggregates and fibers as ellipsoids and cylinders, respectively, the effect of the features of aggregates and fibers (e.g., aggregate fraction, gradation, fiber length, etc.) in high performance concrete (HPC) on the percolation of ITZ around them is investigated [28], and the results show that although the ITZ regions in HPC are thinner and not percolated, they can be re-percolated by the addition of just a few fibers. According to the above studies [26,27,28] for the granular system composed of poly-shaped particles here, it can be found that the variation of ITZ percolation in them would be much more complicated than in the mono-shaped particle systems, which also indirectly proves the necessity of distinguishing the geometric difference between coarse aggregates and fine aggregates in the numerical prediction of ITZ percolation in the concrete systems.

In order to overcome these two simplistic assumptions mentioned above and determine the ITZ percolation associated with the polydispersity of aggregate shapes and size gradations, the coarse aggregates and fine aggregates in concrete are respectively assumed to be polygons and ovals based on their real morphologies in Figure 1. Moreover, the size gradations of them are also expressed by two separate functions instead of a single contiguous function as described in refs. [15,16,18,19,20,21,22,23,24]. On the basis of above two preconditions, the sensitivity of ITZ percolation to the features of both coarse aggregates and fine aggregates in a more realistic way is studied in this paper. Besides, it should be stressed that to reduce the research difficulty and shorten the computation time, the whole work here is conducted in the two-dimensional (2D) case.

The details of this paper are arranged as follows: first, the mathematical expressions of both ovals and regular polygons, the modeling of uniform ITZ around them, and the generating procedure of 2D meso-scale models of concrete are successively presented in Section 2. By combining these models with continuum percolation theory, the effective percolation of the uniform ITZ around both oval fine aggregates and polygonal coarse aggregates is explicitly simulated in Section 3, and the effect of the polydispersity of aggregate shapes and sizes (e.g., the aggregate shape, gradation, and sand ratio) on the critical threshold *ϕ*_agg,c_ is quantitatively evaluated in detail then. Based on the simulated results of *ϕ*_agg,c_, an empirically analytical expression is further proposed by the linear regression analysis, and the comparisons of the predicted value here and the numerical results of the existing function are then presented. Finally, some interesting conclusions are highlighted in Section 4.

## 2. Modeling and Methods

### 2.1. Meso-Scale Models of Concrete Containing Ovals and Polygons

#### 2.1.1. Geometric Expressions of Ovals and Polygons

**Figure 2:**
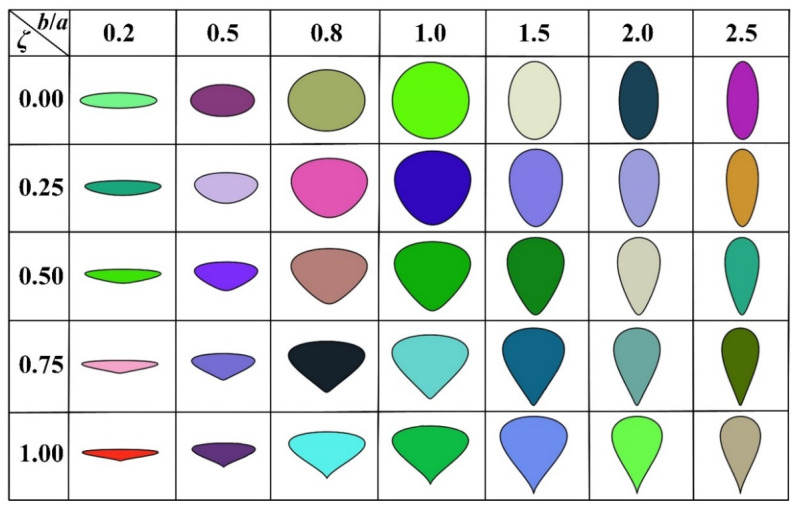
The geometric shapes of ovals with different aspect ratio *b*/*a* and tapering coefficient *ζ*.

The oval is a catch-all term for a family of 2D non-centrosymmetric particles that include circles, ellipses, egg-shaped particles, and other convex-shaped particles with smooth contours, as shown in Figure 2. It has been widely used to characterize the essential features of components in materials [29,30] due to its extensive representation. Based on the geometric features of the oval particles in Figure 2, the profile of ovals can be simply treated as an extension of ellipses, and their mathematical expression can be described by Equation (1).

(1) $$\frac{x^{2}}{{\left(\zeta \frac{a}{b}y+a\right)}^{2}}+\frac{y^{2}}{b^{2}}=1.0\;,$$

where *ζ* is a tapering coefficient that varies from 0.0 to 1.0, *a* and *b* are the semi-axis lengths of the oval in the *x*- and *y*-axis directions, respectively, and the ratio of *b* to *a* is defined as the aspect ratio of the oval.

**Figure 3:**
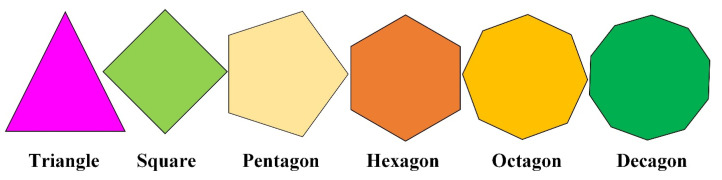
The geometric shapes of regular polygons with different number of edges.

As shown in Figure 3, a set of regular polygons with different numbers of edges are graphically displayed. According to the study in [18], the geometric topographies of these polygons can be mathematically expressed via the collection of the subsets of vertices and edges. Take the regular pentagon as an example; it is assumed that the centroid of the particle is located at the origin of rectangle coordinates and the *x*-axis passes through one of its vertices. The size and length of the pentagon are denoted by the notation *l*. Furthermore, all the vertices P_i_ (i.e., P_1,_ P_2,_ P_3,_ P_4,_ and P_5_) of a particle can be symbolized by Equation (2).

(2) $$P_{i}=\left\{ \begin{array}{l} P_{1}={\left(\frac{l}{2}\csc\left(\frac{\pi }{5}\right),0\right)}^{T},P_{2}={\left(\frac{l}{2}\csc\left(\frac{\pi }{5}\right)\cos\left(\frac{2\pi }{5}\right),l\cos\left(\frac{\pi }{5}\right)\right)}^{T},\\ P_{3}={\left(-\frac{l}{2}\cot\left(\frac{\pi }{5}\right),\frac{l}{2}\right)}^{T},P_{4}={\left(-\frac{l}{2}\cot\left(\frac{\pi }{5}\right),-\frac{l}{2}\right)}^{T},\\ P_{5}={\left(\frac{l}{2}\csc\left(\frac{\pi }{5}\right)\cos\left(\frac{2\pi }{5}\right),-l\cos\left(\frac{\pi }{5}\right)\right)}^{T}. \end{array}\right.$$

Moreover, the five edges of a pentagon are also symbolized by the notation L_i_ (i.e., L_1,_ L_2,_ L_3,_ L_4,_ and L_5_), and their detailed expressions can be further mathematically described as: L_1_ = {P_1_, P_2_}, L_2_ = {P_2_, P_3_}, L_3_ = {P_3_, P_4_}, L_4_ = {P_4_, P_5_} and L_5_ = {P_5_, P_1_}. The relevant geometric information for other kinds of regular polygons has been presented in [18] and is not elaborated again.

#### 2.1.2. Modeling of ITZ around Complex-Shaped Aggregates

In the field of material science, the importance of interphase thickness has been widely acknowledged. For the concrete systems, the thickness of ITZ *t*_ITZ_ around aggregate particles generally fluctuates in a certain range (e.g., [15 μm, 50 μm] in [31], [50 μm, 200 μm] in [32], [100 μm, 200 μm] in [33], etc.), and its specific value may also be differential at different locations around a single aggregate. Strictly speaking, the values of ITZ thickness in concrete would be closely conditioned by a variety of factors such as the composition, test method, curing condition, mixture ratio, production technology, and so on, which is deemed to be a very complicated issue. However, to accurately describe the quantitative relationship between the ITZ thickness and the structural features (or properties) of materials, the ITZ thickness around the aggregates is generally assumed to be a constant in the numerical modeling of concrete. As the relationship between them is formulized, the corresponding expression can be directly applied by substituting the statistical average of ITZ thickness in real material into it. In this study, the commonly used assumption mentioned above is also adopted for the sake of simplicity. That is to say, all the ITZs in the models are assumed to be the uniform interfacial layers coated on the surface of particles, and the overlap of them can freely occur.

To generate the ITZ with a uniform thickness, various techniques were developed based on the geometric shapes of particles. For the ovals, the outer contour of ITZ around them can be discretized into a limited number of points (i.e., (*x*_ITZ_, *y*_ITZ_)), and the mathematical expression of these discrete points can be further derived by combining the generic formula of ovals (i.e., Equation (1)) and the unit normal vectors (**n***_x_*, **n***_y_*) of the points on the contour of these particles as follows:

(3) $$\frac{\partial F\left(x,y\right)}{\partial x}=\frac{2x}{{\left(\zeta \frac{a}{b}y+a\right)}^{2}}\;,$$

(4) $$\frac{\partial F\left(x,y\right)}{\partial y}=\frac{-2a\zeta x^{2}}{b{\left(\zeta \frac{a}{b}y+a\right)}^{3}}+\frac{2y}{b^{2}}\;,$$

(5) $$\left(n_{x},n_{y}\right)=\left\{\frac{\partial F}{\partial x}/\sqrt{{\left(\frac{\partial F}{\partial x}\right)}^{2}+{\left(\frac{\partial F}{\partial y}\right)}^{2}},\frac{\partial F}{\partial y}/\sqrt{{\left(\frac{\partial F}{\partial x}\right)}^{2}+{\left(\frac{\partial F}{\partial y}\right)}^{2}}\right\}\;,$$

(6) $$\{\begin{array}{c} x_{\mathrm{ITZ}}=x+n_{x}\cdot t_{\mathrm{ITZ}}\;,\\ y_{\mathrm{ITZ}}=y+n_{y}\cdot t_{\mathrm{ITZ}}\;, \end{array}\;$$

where *t*_ITZ_ is the thickness of the uniform ITZ around aggregates, (*x*, *y*) represent the spatial coordinates of the points on the contour of the oval.

For the regular polygons, the outer contour of ITZ around them can be generally subdivided into the part of vertices and the part of edges, as described in [18]. The part of the vertices on the contour of ITZ can be viewed as the circular arcs, which are composed of a limited number of discrete points at a fixed distance from the vertexes. The part of the edges on the contour of ITZ can be treated as the line segments, which are parallel to the original edges of the polygon, and they can be constructed by translating the original edges by a preset distance along the normal line of these edges.

#### 2.1.3. Generation of the 2D Meso-Scale Models of Concrete

**Figure 4:**
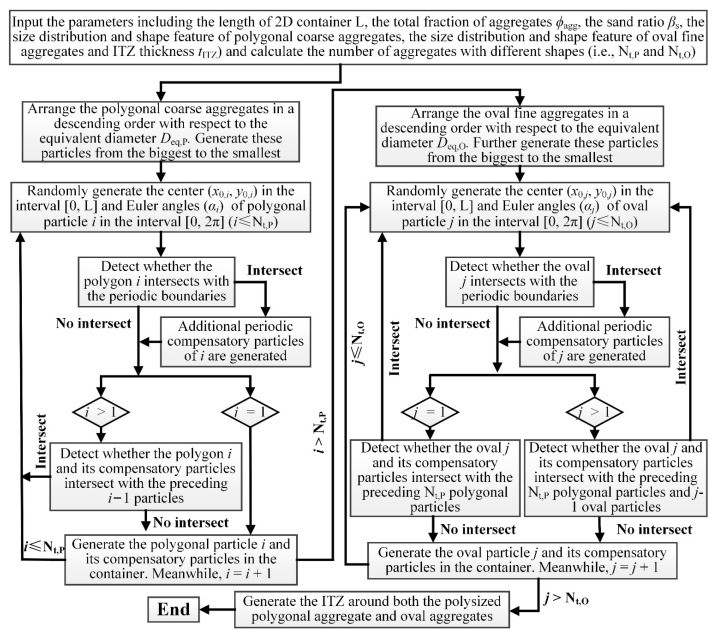
The flowchart of the procedure for generating the 2D models of polyphase concrete.

Actually, both coarse aggregates and fine aggregates in concrete are assemblies of polysized particles. To ascertain the influence of aggregate size distributions on the features of ITZ around them, two common functions (i.e., the EVF function and Fuller function described by Equation (7)) used in the modeling of concrete materials are adopted here to characterize the size gradation (PSG) of these aggregate particles by reference to the literature [34]. Moreover, to uniformly symbolize the sizes of these non-spherical particles with different sizes, the equivalent diameter *D*_eq_, which is defined as the diameter of a circle having the same area as that of a 2D complex-shaped particle, is further introduced. Finally, by combining a series of the above preconditions with the RSP algorithm in Figure 4, meso-scale models of polyphase concrete composed of oval fine aggregates, polygonal coarse aggregates, uniform ITZ, and homogeneous cement matrix can be generated in 2D space. The detailed algorithms and technique have been presented in previous studies [18,29,34,35,36,37]. To avoid duplication, they are not stated in detail in this paper.

(7) $$F_{A}\left(D_{\mathrm{eq}}\right)=\{\begin{array}{cc} \frac{D_{\mathrm{eq}}^{1/2}-D_{\mathrm{eq},\min}^{1/2}}{D_{\mathrm{eq},\max}^{1/2}-D_{\mathrm{eq},\min}^{1/2}} & \;\mathrm{Fuller}\;\mathrm{distribution},\\ \frac{\ln\left(D_{\mathrm{eq}}\right)-\ln\left(D_{\mathrm{eq},\min}\right)}{\ln\left(D_{\mathrm{eq},\max}\right)-\ln\left(D_{\mathrm{eq},\min}\right)} & \mathrm{EVF}\;\mathrm{distribution}, \end{array}$$

where *F_A_*(*D*_eq_) is the area-based cumulative probability function, *D*_eq,min_ and *D*_eq,max_ are the minimum and maximum equivalent diameters of particles, respectively.

**Figure 5:**
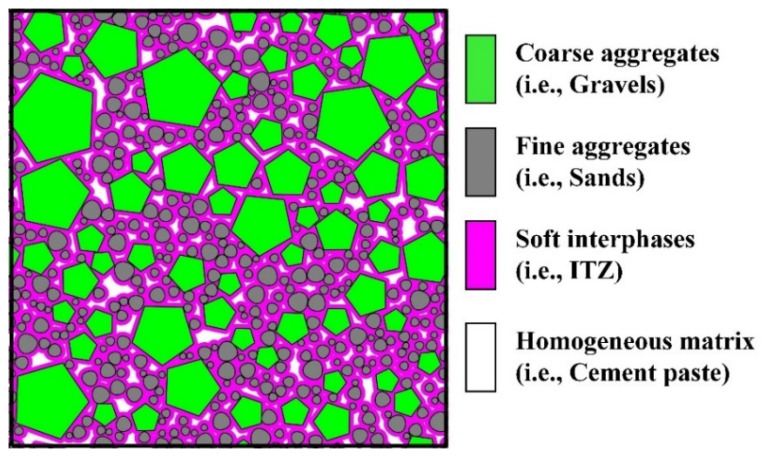
The meso-scale model of polyphase concrete composed of pentagonal coarse aggregates, oval fine aggregates, ITZ and homogeneous matrix (*D*_eq_ = Fuller 1.0–4.5 mm for ovals, *D*_eq_ = Fuller 5–20 mm for regular polygons).

As shown in Figure 5, taking the Fuller function in Equation (7) as an example, one sample of the 2D model of concrete comprising of pentagonal coarse aggregates, oval fine aggregates, uniform ITZ, and cement matrix with the periodic boundary conditions is displayed graphically. In this model, the size length L of a square container is equal to 100 mm. The total fraction *ϕ*_agg_ of aggregate particles is 0.75, and the sand ratio *β*_s_ (i.e., the area ratio of fine aggregates to the sum of both coarse aggregates and fine aggregates) is 0.40. Besides, all the values of *t*_ITZ_ around the aggregates are set to 100 μm.

### 2.2. Percolation Simulation of ITZ around Polydisperse Aggregates

**Figure 6:**
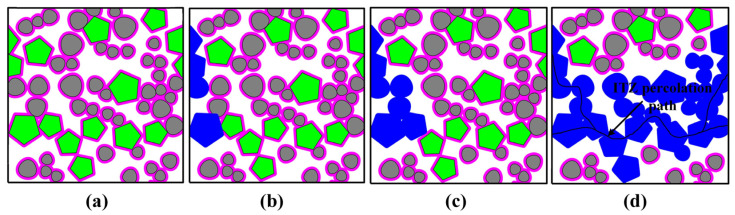
Schematic diagram of the searching procedure for the ITZ percolation paths around both oval fine particles and polygonal coarse particles: (**a**) The generation of 2D polyphase model; (**b**) The identification of the particles that can intersect with the left edge; (**c**) The identification of the particles that intersect with the labeled particles; (**d**) The realization of the ITZ percolation path.

As of now, a wide variety of percolation models with HCSS networks and numerical techniques (e.g., forest fire models, tree-burning algorithms, etc.) have been developed recently [7,9,15,17,18,19,26]. In these HCSS models, the criticality of the emergence of the connected paths is generally represented by the critical threshold, which is commonly symbolized by the critical fraction *ϕ*_agg,c_ of particulate components. To quantify the ITZ percolation around the mixture of ovals and polygons, a commonly used numerical method in ref. [15] is also adopted in this study, and the detailed procedure for searching the percolation path of ITZ is shown in Figure 6. Firstly, the 2D polyphase model with the periodic boundary condition is generated, as shown in Figure 6a. By taking the left and right edges of the container as an example, the first step is to search for the aggregate particles around which the ITZs can intersect with the left edge and label them with a new tag (i.e., the blue in Figure 6b). Afterward, search further for the aggregate particles around which the ITZs can intersect with the ITZs around the preceding labeled particles and label them with the same tag, as shown in Figure 6c. The above process is performed iteratively until there are no additional particles. Finally, judge whether or not the ITZs around these labeled aggregates can intersect with the right edge of the container. If there exists the interconnectivity among ITZs throughout the whole system, as shown in Figure 6d, the ITZ percolation is deemed to occur in this sample.

In light of the independence of each sample, the occurrence of ITZ percolation in the finite-sized system can be regarded as a Bernoulli probability variable. For a large number of trials, the number of successes could be mathematically represented by the binominal distribution, and the number ratio of the percolating samples N_pcl_ to the total samples N_total_ (i.e., P_ITZ_ = N_pcl_/N_total_) is generally defined as the percolation probability P_ITZ_. A great number of studies [13,15,18,19,37] have clearly shown that the stability of P_ITZ_ is closely dependent on the value of N_total_. To improve the statistical stability of P_ITZ_, N_total_ is set to be 2000 here by referring to the description in [18]. Furthermore, as the probability P_ITZ_ for the finite-sized systems with different *L* and *ϕ*_agg_ is obtained, the critical percolation threshold *ϕ*_agg,c_ can be further determined by the curve-fitting method with the following formula (i.e., Equation (8)).

(8) $$P_{\mathrm{ITZ}}\left(\phi_{\mathrm{agg}},L\right)=\frac{1}{2}\cdot \left\{1+\mathrm{erf}\left[\frac{\phi_{\mathrm{agg}}-\phi_{\mathrm{agg},c}}{\Delta \left(L\right)}\right]\right\}\;,$$

where *ϕ*_agg,_ is the relative content of aggregate particles, *ϕ*_agg,c_ is the critical percolation threshold of ITZ, and Δ(*L*) is the percolation transition width.

According to the study by Pan et al. [26], the size length L of percolation models would mainly affect the value of Δ(*L*), but has no great influence on the critical percolation threshold *ϕ*_agg,c_. By reference to the findings (i.e., *L* ≥ 5*D*_eq,max_) in [19] and the criterion for determining the representative volume element (REV) in [38] (i.e., the RVE size should be at least 3~5 times of the maximum particle diameter), the size lengths of all the generated models here are set to not less than 5 times of the maximum size *D*_eq,max,P_ of polygonal coarse aggregates (i.e., *L*/*D*_eq,max,P_ ≥ 5.0).

## 3. Results and Discussion

In the section, a series of parametric analyses about the effect of aggregate features on the critical threshold *ϕ*_agg,c_ of ITZ percolation are conducted. Based on the simulated results of *ϕ*_agg,c_, an approximately analytical function is further developed to fast evaluate the ITZ percolation in the 2D case of polyphase composite systems.

### 3.1. Effect of Particle Shapes on the ITZ Percolation

**Figure 7:**
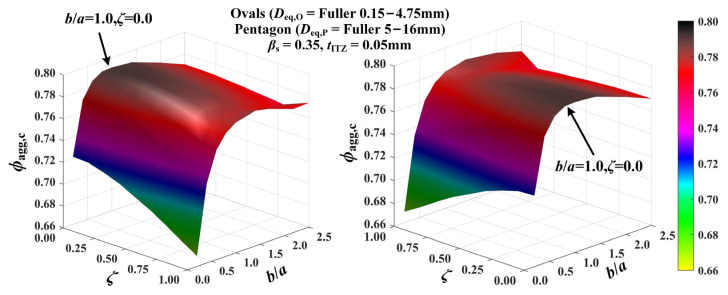
Effect of the geometric shapes of oval particles on the ITZ percolation.

By assuming the coarse aggregates as regular pentagons, the sensitivity of the critical threshold *ϕ*_agg,c_ to the shape of oval fine aggregates is studied first in Figure 7. The curved surfaces clearly show that the threshold *ϕ*_agg,c_ is an apparently *ζ*- and *b*/*a*-dependent argument, which possesses the obviously different trends with the parameters *ζ* and *b*/*a*. On the one hand, as *ζ* increases from 0.0 to 1.0, *ϕ*_agg,c_ basically shows a slightly decreasing trend under a constant *b*/*a*. On the other hand, when the coefficient *ζ* is fixed, all the *ϕ*_agg,c_-*b*/*a* curves could be subdivided into two stages (i.e., the upward stage and the downward stage) with the increase of *b*/*a* in [0.2, 2.5], and the inflection points are basically located at *b*/*a* = 1.0. In the upward stage, the value of *ϕ*_agg,c_ increases rapidly with the increasing *b*/*a* from 0.2 to 1.0 in the parabolic manner. However, with the further growth of *b*/*a*, *ϕ*_agg,c_ presents a very slowly descending trend. Overall, the maximal threshold *ϕ*_agg,c_ appears at *ζ* = 0.0 and *b*/*a* = 1.0, which indicates that the ITZ percolation paths are least likely to be formed if the fine aggregates are assumed to be circular. In [29], the variation curves of the percolation threshold *ε_c,mm_* of porous systems composed of oval pores with *b*/*a* in [0.2, 5.0] and *ζ* in [0.0, 0.5] were presented. By comparison, one can clearly see that the variation of *ϕ*_agg,c_ with *b*/*a* and *ζ* here is commendably consistent with these results. These comparisons indirectly indicate the reliability of the variation of *ϕ*_agg,c_.

**Figure 8:**
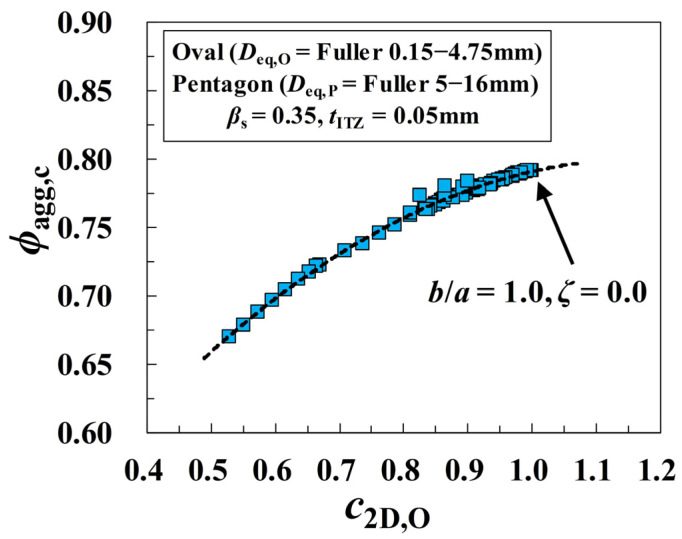
Sensitivity of the critical threshold *ϕ*_agg,c_ in Figure 7 to the circularity *c*_2D,O_ of oval particles.

In the literature [18], the aggregate shapes are uniformly symbolized by the circularity *c*_2D_ (i.e., the perimeter ratio of circles to non-circular particles having the same area), and the critical threshold *ϕ*_agg,c_ is formulized as a function of *c*_2D_. By referring to this strategy, the circularities *c*_2D,O_ of ovals with *b*/*a* in [0.2, 2.5] and *ζ* in [0.0, 1.0] are calculated, and the sensitivity of *ϕ*_agg,c_ to the circularity *c*_2D,O_ is further graphically presented based on the results of *ϕ*_agg,c_ in Figure 7. It can be seen that all the data points in Figure 8 are roughly converging to a quadratic increasing curve, which means that the smaller the circularity *c*_2D,O_ of fine aggregates is, the more easily the percolation paths of ITZ around them are formed. The above trend indicates that the circularity can be well used to represent the quantitative relationship between the ITZ percolation threshold and the oval shapes. This provides the foundation for the derivation of the analytical model of *ϕ*_agg,c_ in the following sections.

**Figure 9:**
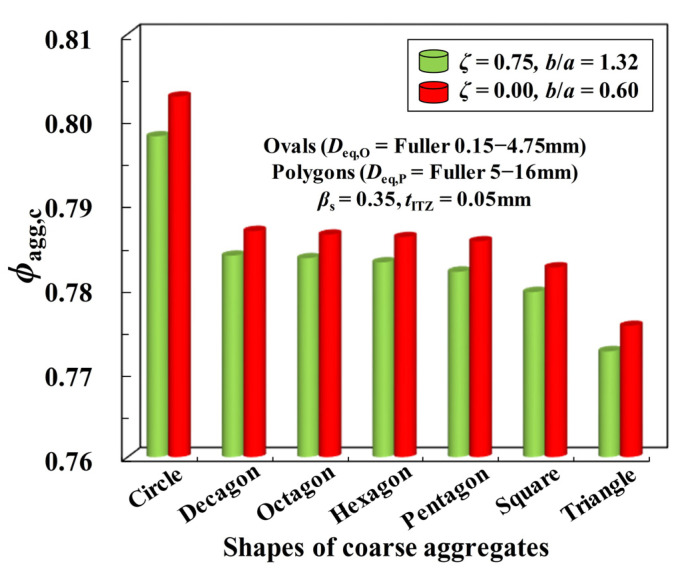
Effect of the geometric shapes of polygonal particles on the ITZ percolation.

In Figure 9, the sensitivity of ITZ percolation to the shape of polygonal coarse aggregates is further studied. The results clearly show that no matter what the shape of fine aggregates is, *ϕ*_agg,c_ always possesses a similar trend with the shape shifting of coarse aggregates (i.e., Circle > Decagon > Octagon > Hexagon > Pentagon > Square > Triangle). By combining with the circularity *c*_2D,P_ of these polygons, the value of *ϕ*_agg,c_ here also shows the downward trend with the increasing *c*_2D,P_ and the attenuation amplitude of *ϕ*_agg,c_ is largely dependent on the relative difference of *c*_2D,P_ for different polygons. However, by contrast with the dataset in [18], it can be found that the impact degree of aggregate shape (i.e, *c*_2D,O_ or *c*_2D,P_) on the value of *ϕ*_agg,c_ here is significantly weakened, which indicates that the analytical formula in the previous paper [18] may not be directly applicable. Overall, the ITZ percolation would be influenced by the synergy of both fine aggregate shapes and coarse aggregate shapes. It is very necessary to distinguish the geometric difference between fine aggregate and coarse aggregate in the modeling of concrete.

### 3.2. Effect of Particle Sizes on the ITZ Percolation

**Figure 10:**
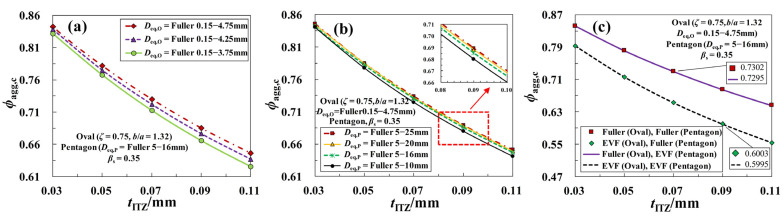
Effect of the size distributions of aggregate particles on the curves of *ϕ*_agg,c_-*t*_ITZ_: (**a**) Effect of the size ranges of oval fine aggregates; (**b**) Effect of the size ranges of polygonal coarse aggregates; (**c**) Effect of the size gradations of aggregates.

Size-polydispersity of particles inevitably exists in most granular materials, and a number of studies about the ITZ percolation involving the polysized aggregates have been conducted recently [13,15,18,19,26], but the size distribution difference between fine aggregates and coarse aggregates is often overlooked. In the section, the dependence of critical threshold *ϕ*_agg,c_ on the size distributions of both fine and coarse aggregates is separately analyzed. Figure 10a,b show the sensitivity of *ϕ*_agg,c_ to the maximum diameter *D*_eq,max,O_ of fine aggregates and maximum diameter *D*_eq,max,P_ of coarse aggregates, respectively. It can be seen that all the curves of *ϕ*_agg,c_-*t*_ITZ_ possess a remarkable inverted parabolic downward trend with the increasing *t*_ITZ_ in [0.03 mm, 0.11 mm]. This is mainly due to the fact that the thicker the ITZ thickness is, the greater the collision probability of ITZ around different aggregates is, which in turn promotes the formation of ITZ percolation paths in concrete. Besides, when the value of *D*_eq,min,O_ or *D*_eq,min,P_ is held constant, the threshold *ϕ*_agg,c_ shows the various degrees of growth with the increase of *D*_eq,max,O_ or *D*_eq,max,P_. That is to say, the smaller the value of *D*_eq,max,O_ or *D*_eq,max,P_ is, the more easily the ITZ percolation paths are formed. By comparison, it is not difficult to find that the dependence of *ϕ*_agg,c_ on the size *D*_eq,max,P_ is significantly lower than the size *D*_eq,max,O_. Hence, for the sake of simplicity, the effect of the size ranges of coarse aggregates on the ITZ percolation can be negligible in some cases.

By using Equation (7), the effect of PSG of both fine aggregates and coarse aggregates on the ITZ percolation is further shown in Figure 10c. One can clearly see that the PSG of fine aggregates has a significant influence on the critical threshold *ϕ*_agg,c_. The value of *ϕ*_agg,c_ for EVF gradation is much smaller than the corresponding threshold for Fuller gradation. This is largely due to the fact that the number of small-sized aggregates in the system for EVF gradation is generally larger than that for Fuller gradation. The above trend of *ϕ*_agg,c_ here is in good agreement with the existing findings for the mono-shaped particle system in [15,18,19,26]. In contrast to that, the PSG of coarse aggregates has little effect on the ITZ percolation around them. This phenomenon has not yet been reported in previous literature and needs to be given enough consideration.

According to the study in [18], the influence of all the size-polydispersities of aggregate particles on the ITZ percolation *ϕ*_agg,c_ can be attributed to the aggregate fineness, which is commonly symbolized by the specific surface area C_A_. Based on this view, the average specific surface area $C_{A}^{\mathrm{avg}}$ of both oval aggregates and polygonal aggregates is derived here to represent the fineness of aggregate particles in concrete, as expressed by Equations (9)–(11).

(9) $$C_{A}^{\mathrm{avg}}=\beta_{s}\cdot C_{A,O}+\left(1-\beta_{s}\right)\cdot C_{A,P}\;,$$

(10) $$C_{A,O}=\frac{\int_{D_{\mathrm{eq},\min,O}}^{D_{\mathrm{eq},\max,O}}C_{O}\left(D_{\mathrm{eq},O}\right)\cdot f_{N}\left(D_{\mathrm{eq},O}\right)dD_{\mathrm{eq},O}}{\int_{D_{\mathrm{eq},\min,O}}^{D_{\mathrm{eq},\max,O}}A_{O}\left(D_{eq,O}\right)\cdot f_{N}\left(D_{\mathrm{eq},O}\right)dD_{\mathrm{eq},O}}\;,$$

(11) $$C_{A,P}=\frac{\int_{D_{\mathrm{eq},\min,P}}^{D_{\mathrm{eq},\max,P}}C_{P}\left(D_{\mathrm{eq},P}\right)\cdot f_{N}\left(D_{\mathrm{eq},P}\right)dD_{\mathrm{eq},P}}{\int_{D_{\mathrm{eq},\min,P}}^{D_{\mathrm{eq},\max,P}}A_{P}\left(D_{\mathrm{eq},P}\right)\cdot f_{N}\left(D_{\mathrm{eq},P}\right)dD_{\mathrm{eq},P}}\;,$$

where C_A,O_ and C_A,P_ are the specific surface areas of oval fine aggregates and polygonal coarse aggregates, C_O_(*D*_eq,O_), A_O_(*D*_eq,O_) and *f_N_*(*D*_eq,O_) are the perimeter, area and number-based probability function for oval fine aggregates, respectively; C_P_(*D*_eq,P_), A_P_(*D*_eq,P_), and *f_N_*(*D*_eq,P_) are the perimeter, area, and number-based probability function for polygonal coarse aggregates, respectively.

For the Fuller function and EVF function in Equation (7), the corresponding number-based probability functions can be expressed as below:

(12) $$f_{N}\left(D_{\mathrm{eq}}\right)=\{\begin{array}{cc} \frac{3}{2D_{\mathrm{eq}}^{5/2}\left(D_{\mathrm{eq},\min}^{-3/2}-D_{\mathrm{eq},\max}^{-3/2}\right)} & \;\mathrm{Fuller}\;\mathrm{distribution},\\ \frac{2}{D_{\mathrm{eq}}^{3}\left(D_{\mathrm{eq},\min}^{-2}-D_{\mathrm{eq},\max}^{-2}\right)} & \mathrm{EVF}\;\mathrm{distribution}. \end{array}$$

By substituting Equation (12) into Equations (10) and (11), the theoretical solution of C_A_ for different types of particles can be further derived, and the detailed formulas (i.e., Equation (13)) are expressed as a function of *c*_2D_.

(13) $$C_{A}\left(c_{2D}\right)=\{\begin{array}{cc} \frac{4\cdot \left(D_{\mathrm{eq},\max}^{-1/2}-D_{\mathrm{eq},\min}^{-1/2}\right)}{c_{2D}\cdot \left(D_{\mathrm{eq},\min}^{1/2}-D_{\mathrm{eq},\max}^{1/2}\right)} & \;\mathrm{Fuller}\;\mathrm{distribution},\\ \frac{4\cdot \left(D_{\mathrm{eq},\max}^{-1}-D_{\mathrm{eq},\min}^{-1}\right)}{c_{2D}\cdot \left[\ln\left(D_{\mathrm{eq},\min}\right)-\ln\left(D_{\mathrm{eq},\max}\right)\right]} & \mathrm{EVF}\;\mathrm{distribution}. \end{array}$$

**Figure 11:**
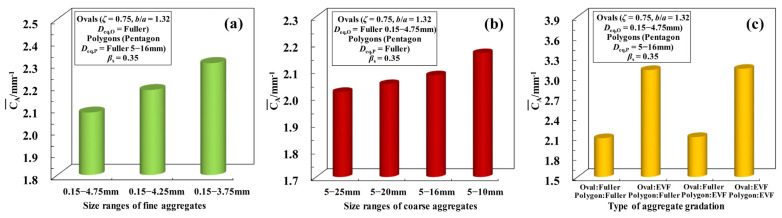
Comparisons of the average specific surface areas of aggregate particles for all the cases: (**a**) The curves in Figure 10a; (**b**) The curves in Figure 10b; (**c**) The curves in Figure 10c.

Finally, by incorporating Equation (13) into Equation (9), the average specific surface area $C_{A}^{\mathrm{avg}}$ of aggregate particles for all the cases in Figure 10 is obtained, and the comparisons of them are shown in Figure 11a–c. From these figures, the following trends can be found: (a) as shown in Figure 11a,b, with the increase of *D*_eq,max,O_ or *D*_eq,max,P_, the value of $C_{A}^{\mathrm{avg}}$ is also increased significantly, and its sensitivity to the size range of fine aggregates is much bigger than that for coarse aggregates; (b) The PSG of coarse aggregates has little influence on the value of $C_{A}^{\mathrm{avg}}$, however, when the PSG of coarse aggregates is fixed, $C_{A}^{\mathrm{avg}}$ for the fine aggregates with EVF gradation is significantly greater than the value of $C_{A}^{\mathrm{avg}}$ with Fuller gradation, as shown in Figure 11c. By combining with the curves in Figure 10, a relatively consistent trend can be provided (i.e., the greater the average specific surface area of aggregate particles is, the smaller the percolation threshold of ITZ *ϕ*_agg,c_ tends to be), which also provides a direction for the derivation of the model of *ϕ*_agg,c_.

### 3.3. Effect of Sand Ratio on the ITZ Percolation

**Figure 12:**
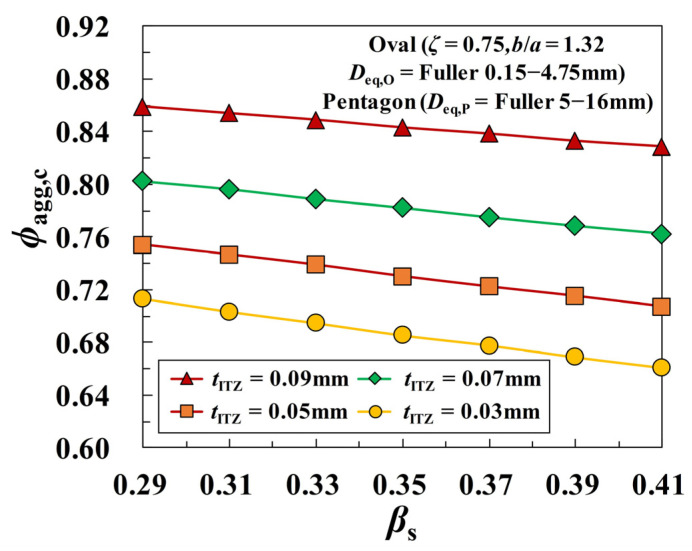
Effect of the sand ratio *β*_s_ on the ITZ percolation under a constant ITZ thickness.

**Figure 13:**
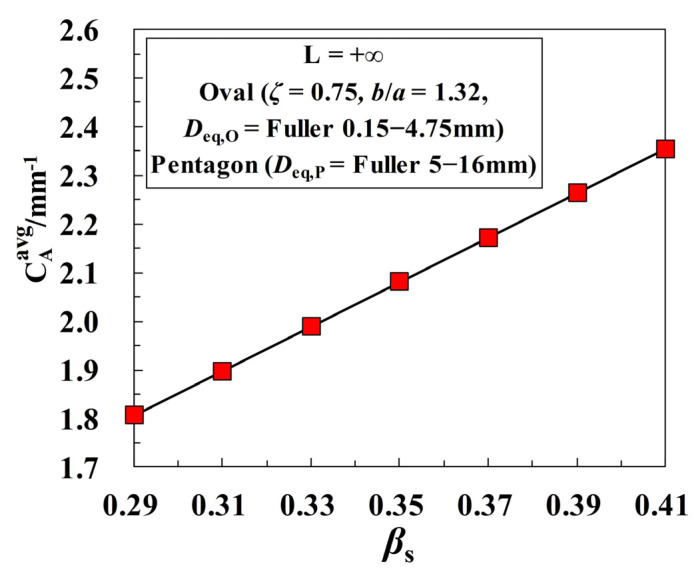
Effect of the sand ratio *β*_s_ on the average specific surface area of particles for the cases in Figure 12.

The sand ratio *β*_s_ is an important factor that affects the workability and mechanical properties of concrete materials. Under the assumption of *D*_eq,O_ = Fuller 0.15–4.75 mm and *D*_eq,P_ = Fuller 5–16 mm, the sensitivity of the critical threshold *ϕ*_agg,c_ to the sand ratio *β*_s_ is investigated in Figure 12. One can see that all the curves of *ϕ*_agg,c_-*β*_s_ show an apparently linear decreasing tendency, which is dependent on the specific thickness of ITZ. That is to say, the greater the proportion of fine aggregates in concrete is, the more easily the ITZ percolation path is formed. Actually, the effect of *β*_s_ on *ϕ*_agg,c_ can also be mapped to the variation of aggregate fineness. As the sand ratio *β*_s_ increases, the number of fine aggregates is also on the rise, which in turn leads to the rapid growth of the average specific surface area of aggregate particles, as shown in Figure 13.

### 3.4. Formulation and Verification

**Figure 14:**
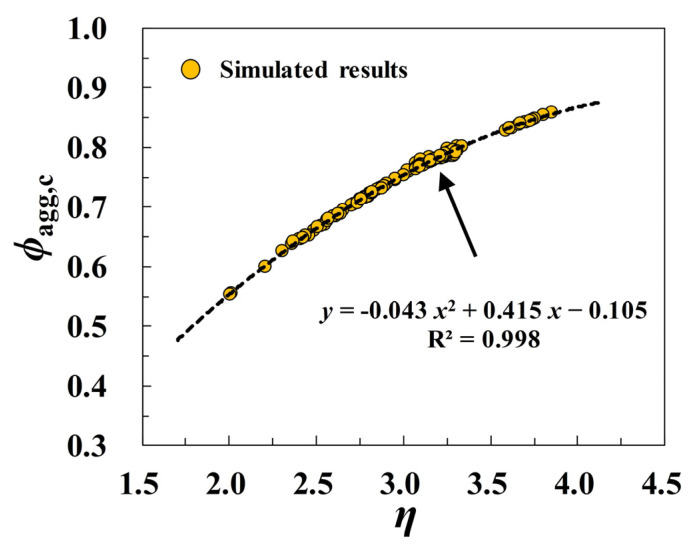
The variation of the critical percolation threshold *ϕ*_agg,c_ as a function of the argument *η*.

Through a series of in-depth analyses and comparisons, it can be found that the ITZ thickness, aggregate fineness, and aggregate shape can be deemed to be the main contributing parameters that affect the ITZ percolation when the synergetic effects of aggregate shapes and aggregate sizes are taken into consideration. To quantitatively characterize the sensitivity of the critical percolation threshold *ϕ*_agg,c_ to all of the aforementioned factors, a new argument *η* (i.e., Equation (14)) is proposed here by referring to the form of the proposed parameter (i.e., −ln(*t*_ITZ_C_A_)) in [18] and all the simulated results of *ϕ*_agg,c_ in Figure 7, Figure 9, Figure 10 and Figure 12 are plotted as a function of *η*, as shown in Figure 14.

(14) $$\begin{array}{l} \eta =-\ln\left\{t_{\mathrm{ITZ}}\left[C_{A,P}\left(1-\beta_{s}\right)+C_{A,O}\beta_{s}\right]\right\}+\left[c_{2D,P}\left(1-\beta_{s}\right)+c_{2D,O}\beta_{s}\right]\\ \;=-\ln\left(t_{\mathrm{ITZ}}C_{A}^{\mathrm{avg}}\right)+\left[c_{2D,P}\left(1-\beta_{s}\right)+c_{2D,O}\beta_{s}\right]\;. \end{array}$$

**Figure 15:**
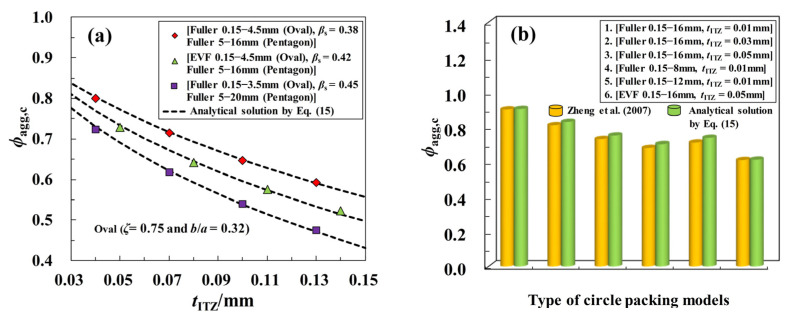
Comparisons of the numerical solutions of *ϕ*_agg,c_ predicted by Equation (15) with (**a**) the simulated results of *ϕ*_agg,c_ in some extra cases; (**b**) the reported results in [15].

The curve of *ϕ*_agg,c_-*η* in Figure 14 clearly shows that *ϕ*_agg,c_ possesses an apparently monotonical uptrend with the increase of *η* in a quadratic manner. By the linear regression analysis method, a numerical fitting formula is obtained in Figure 14, and the approximately empirical expression of *ϕ*_agg,c_ (i.e., Equation (15)) can be further derived by incorporating the argument *η* into this quadratic formula. To validate the reliability of the proposed function of *ϕ*_agg,c_, the comparison of the simulated values of *ϕ*_agg,c_ in some extra cases with the predicted approximation obtained by Equation (15) is shown in Figure 15a. It can be seen that all the curves of *ϕ*_agg,c_-*t*_ITZ_ here are in excellent agreement with the simulated results. Figure 15b further displays the quantitative comparison of the theoretical solution of *ϕ*_agg,c_ with the numerical results reported by Zheng et al. [15], and the approximate agreement between them can also be observed clearly. All these comparisons suggest that the proposed analytical formula of *ϕ*_agg,c_ can reproduce the results of *ϕ*_agg,c_ with the satisfying accuracy, and it can be well used to quickly evaluate the ITZ percolation in 2D polyphase systems.

(15) $$\begin{array}{ll} \phi_{\mathrm{agg},c}= & -0.043{\left\{\ln\left(t_{\mathrm{ITZ}}C_{A}^{\mathrm{avg}}\right)-\left[c_{2D,P}\left(1-\beta_{s}\right)+c_{2D,O}\beta_{s}\right]\right\}}^{2}-\\ & \;\;\;0.415\left\{\ln\left(t_{\mathrm{ITZ}}C_{A}^{\mathrm{avg}}\right)-\left[c_{2D,P}\left(1-\beta_{s}\right)+c_{2D,O}\beta_{s}\right]\right\}-0.105\;\;\;\left(R^{2}=0.998\right)\;. \end{array}$$

**Figure 16:**
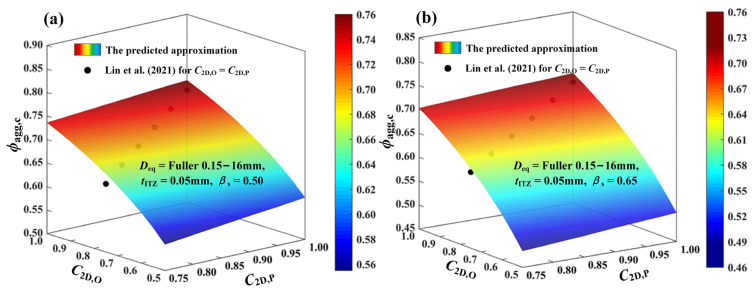
Comparisons of the predicted results of *ϕ*_agg,c_ in the work with the numerical results of *ϕ*_agg,c_ predicted by the formula in [18] (**a**) *β*_s_ = 0.50; (**b**) *β*_s_ = 0.65.

As shown in Figure 16, it is assumed that the ITZ thickness *t*_ITZ_ around aggregates is fixed to be 0.05 mm. The size distributions of aggregate particles are respectively set to *D*_eq_ = Fuller 0.15–10 mm and *D*_eq_ = Fuller 0.15–16 mm. As we know from the calculation, the corresponding sand ratio *β*_s_ in them can be separately equal to 0.50 and 0.65. Take the above cases as the study object, and the predicted approximation of *ϕ*_agg,c_ by the proposed functions (i.e., Equations (9), (13) and (15)) here is presented, and compared with the analytical results predicted by the formula in [18]. It can be seen that with the decrease of *c*_2D,O_ in [0.5, 1.0] or *c*_2D,P_ in [0.75, 1.0], the critical threshold of ITZ *ϕ*_agg,c_ also decreases apparently. Moreover, when the circularities of both fine aggregates and coarse aggregates are identical (i.e., *c*_2D,O_ = *c*_2D,P_), the proposed functions generally provide slightly larger results of *ϕ*_agg,c._ than the results in [18], and the smaller the particle circularity is, the larger the relative error between them would be. This can be blamed on two main reasons. The first one is that the analytical formula in [18] was mainly derived by analyzing the large bodies of data for the ITZ percolation around circles with its size less than 5 mm and the small amount of data for the models of polygons. Literally, the published expression may be more applicable for the 2D mortar systems that composed the circular aggregates. Once it is extended to the concrete systems, including the non-circular aggregates with sizes larger than 5 mm, its effectiveness and accuracy may be limited to some extent. The second reason is that, compared with the existing literature, the size gradations of both coarse aggregate and fine aggregates in this work are separately expressed, which is more practical than the single function. Besides, the size ranges of aggregate particles used in this work are also much larger and wider than before. Simultaneously, some other important factors (e.g., the aggregate shapes and sand ratio) are also considered in this work. All of the above conditions make the predicted results here more accurate and comprehensive. Through the above figures, it can also be seen that the proposed model here is applicable not only to the granular systems of mono-shaped particles (*c*_2D,O_ = *c*_2D,P_), but also to the systems involving the effect of the shape diversity (*c*_2D,O_ ≠ *c*_2D,P_)and relative content (*β*_s_) of different particles. Overall, the model here is bound to have a much broader range of applications in the 2D case compared with the published formula from the previous paper.

According to the curves of *ϕ*_agg,c_ in Figure 7, Figure 8, Figure 9, Figure 10, Figure 11, Figure 12, Figure 13 and Figure 14, it can be found that all the aforementioned factors including ITZ thickness (*t*_ITZ_), aggregate fineness ($C_{A}^{\mathrm{avg}}$) and aggregate shapes (i.e., *c*_2D,P_ and *c*_2D,O_) can be deemed to the contributing factors for the percolation properties of ITZ around the 2D poly-shaped poly-sized aggregates. However, given that all the work in this paper is conducted based on the 2D simplified granular systems, the proposed formulas here can only be applicable to the 2D granular structures. That is to say, it is still difficult to be directly applied in engineering. But, the variation trends of the ITZ percolation with the influencing factors should be basically consistent in both the 2D and 3D cases. Hence, the work in this paper can still play a positive role in the development of similar research in 3D composite systems.

Finally, strictly speaking, the geometric features of aggregate particles in actual concrete materials are much more complex and diverse than that in the models here. By reference to the proposed formula (i.e., Equation (15)) here, an analytical formula of ITZ percolation for the more complex systems is preliminarily suggested, as expressed by Equation (16). The specific values of the parameters (i.e., *a*_1_, *a*_2_, and *a*_3_) and the feasibility of Equation (16) need to be further studied. It is hoped that this hypothesis may provide some help for others in their research.

(16) $$\phi_{\mathrm{agg},c}=a_{1}\cdot {\left[\ln\left(t_{\mathrm{ITZ}}\sum_{i=1}^{N}C_{A,i}\cdot \beta_{i}\right)-\sum_{i=1}^{N}c_{2D,i}\cdot \beta_{i}\right]}^{2}+a_{2}\cdot \left[\ln\left(t_{\mathrm{ITZ}}\sum_{i=1}^{N}C_{A,i}\cdot \beta_{i}\right)-\sum_{i=1}^{N}c_{2D,i}\cdot \beta_{i}\right]+a_{3}\;,$$

where *a*_1_, *a*_2_ and *a*_3_ are three coefficients, C_A,*i*_ and *c*_2D,*i*_ are the specific surface area and circularity of the *i*th types of particles, respectively, *β_i_* is defined as the relative fraction of the *i*th types of particles and the sum of *β*_1_ + *β*_2_ + … *β_i_* … + *β*_N−1_ + *β*_N_ is exactly equal to 1.0, in which N is the total number of particle species in the system.

## 4. Conclusions

In this paper, the sensitivity of ITZ percolation to the features of both coarse aggregates and fine aggregates is studied in a 2D case. To highlight the effect of the shape and size difference between coarse aggregates and fine aggregates in a more realistic way, they are respectively simplified as polygons and ovals here based on the real morphologies of these aggregates. Moreover, the size gradations of them are also expressed by two separate functions instead of the single contiguous function in the literature. By coupling the generated 2D polyphase models of concrete systems with the continuum percolation, the percolation behavior of the uniform ITZ around aggregates is simulated and the effect of the polydispersity of aggregate shapes and sizes on the critical threshold *ϕ*_agg,c_ is quantitatively analyzed, respectively. Numerical variation of the threshold *ϕ*_agg,c_ reveals that: (a) the ITZ thickness (*t*_ITZ_), average aggregate fineness ($C_{A}^{\mathrm{avg}}$), coarse aggregate shape (*c*_2D,P_), and fine aggregate shape (*c*_2D,O_) are four important factors to the ITZ percolation, (b) the sand ratio *β*_s_ is also an important parameter to the ITZ percolation and its effect can be mapped to the aggregate fineness (i.e., the larger the ratio *β*_s_ is, the larger the average specific surface of aggregates would be, which in turn leads to the smaller values of *ϕ*_agg,c_).

By adopting the linear regression analysis, an approximately analytical expression of *ϕ*_agg,c_ (i.e., Equations (9), (13) and (15)) is further proposed and its applicability is verified by comparing with the numerical results, which indicates that the proposed formulas here can reproduce the results of *ϕ*_agg,c_ with the satisfying accuracy. Moreover, by comparing the proposed model with the existing prediction expression, it can be found that the model can produce slightly larger results for the ITZ percolation threshold *ϕ*_agg,c_, which may be closer to reality. Finally, it can not only be applied to the mortar or concrete but also to some other similar granular systems in the 2D case.

## Data Availability

The relevant data can be available upon request by contact with the corresponding authors.

## Nomenclature

*a*, *b*	semi-axis lengths of oval in x- and y-direction, respectively	*ϕ*_agg,c_	critical aggregate fraction (i.e., the critical percolation threshold)
*ζ*	tapering coefficient	*ϕ*_agg_	fraction of aggregate particles
*l*	size length of polygon	Δ(*L*)	percolation transition width
P_i_	ith vertex of polygon	*c*_2D_	circularity of particle
L_i_	ith edge of polygon	*c*_2D,O_, *c*_2D,P_	circularity of oval and polygon, respectively
*t*_ITZ_	thickness of ITZ around aggregate particles	*D*_eq,max,O_	maximum equivalent diameter of oval
**n** *_x_*	*x*-axis component of unit normal vector	*D*_eq,min,O_	minimum equivalent diameter of oval
**n** *_y_*	*y*-axis component of unit normal vector	*D*_eq,max,P_	maximum equivalent diameter of polygon
*β*_s_	sand ratio	*D*_eq,min,P_	minimum equivalent diameter of polygon
*F_A_*	area-based cumulative probability function	C_A_	specific surface area of particle
*D*_eq_	equivalent diameter of particle	C_A,O,_ C_A,P_	specific surface area of ovals and polygons, respectively
*D*_eq,max_	maximum equivalent diameter of particle	C_A,i_	specific surface area of the ith type particle
*D*_eq,min_	minimum equivalent diameter of particle	$C_{A}^{\mathrm{avg}}$	average surface area of both fine and coarse aggregate particles
*D*_eq,O_	equivalent diameter of oval	C_O_, A_O_	perimeter and area of oval, respectively
*D*_eq,P_	equivalent diameter of polygon	C_P_, A_P_	perimeter and area of polygon, respectively
N_pcl_	number of the percolating samples	*f* *_N_*	number-based probability function of particles
N_total_	number of the total samples	*β*_i_	fraction of the ith types of particles
P_ITZ_	percolation probability	*a*_1_*, a*_2_, *a*_3_	three coefficients
*L*	size length of 2D square models		
